# Effects of Facial Threading on Female Skin Texture: A Prospective Trial with Physiological Parameters and Sense Assessment

**DOI:** 10.1155/2019/1535713

**Published:** 2019-08-28

**Authors:** Li-Ying Lin, Shang-Chia Chiou, Shu-Hui Wang, Ching-Chi Chi

**Affiliations:** ^1^Department of Cosmetic Science, Chung Hwa University of Medical Technology, Tainan, Taiwan; ^2^Graduate School of Design, National Yunlin University of Science and Technology, Yunlin, Taiwan; ^3^Department of Dermatology, Far Eastern Memorial Hospital, New Taipei, Taiwan; ^4^Graduate Institute of Applied Science and Engineering, College of Science and Engineering, Fu Jen Catholic University, New Taipei, Taiwan; ^5^Department of Dermatology, Chang Gung Memorial Hospital, Linkou, Taoyuan, Taiwan; ^6^College of Medicine, Chang Gung University, Taoyuan, Taiwan

## Abstract

**Background:**

Facial threading is a common tradition in Taiwan, Southeast Asia (called “Bande Abru”), Middle East (called “Khite”), and Egypt (called “Fatlah”). In addition to the ability to remove facial vellus hairs, facial threading can make the skin fairer and shinier. However, there has been a lack of hard evidence regarding the effects of facial threading on the skin.

**Objective:**

To examine the effects of facial threading on skin physiology as well as visual and touch senses by using scientific instruments.

**Methods:**

A total of 80 participants were allocated to receive facial threading, application of powder only, exfoliation, and shaving. Prior to and following the assigned treatment, a noninvasive skin condition detection device was used to measure skin coarseness, hydration, melanin, and erythema index. Sense assessment and image analysis were also performed.

**Results:**

This study showed that facial threading was found to improve the facial skin roughness indices with significant decreases by 30.4%, 35.9%, and 16.7%, respectively, for the participants' forehead, cheek, and mouth corner skin. No significant adverse changes in moisture levels and skin pigment indices were detected. In addition, there was improvement in subjects' touch sense of their skin and feelings about skin color.

**Conclusions:**

Traditional facial threading can remove facial vellus hairs and lower skin roughness levels, thereby improving the skin texture. However, pricking sensation appeared during the facial threading process, which might cause concerns about irritation.

## 1. Introduction

Most traditional societies believe body hairs in women are indecent and aesthetically unappealing. Women therefore use various methods to remove excessive hairs from their armpits, crotches, arms, and calves. For example, there were records from the Meiji Period in Japan documenting use of razors by women to remove hairs and shape their eyebrows [1]. Moreover, there was an occupation referred to as “顏丿毛拔” (Wan Mian) during the colonial period when Taiwan was ruled by Japan [2]. Nowadays, women can have hairs permanently removed by laser surgery [3].

Facial threading is a common tradition in Taiwan, Southeast Asia (called “Bande Abru”), the Middle East (called “Khite”), and Egypt (called “Fatlah”), to remove vellus hairs on the face [4]. Facial threading uses mechanics and the lever principle [5]. The fine facial hairs are removed via friction caused by yarn rubbing against the skin.

It is a common traditional belief in Taiwan that, in addition to being able to remove the vellus hairs on the face, facial threading can make the skin fairer and shinier. However, there has been a lack of hard evidence regarding the effects of facial threading on the skin. The objective of this prospective trial was to examine the effects of facial threading on skin physiology by utilizing modern scientific instruments. This study also assessed attributes of psychological aspects through touch, visual, and other senses.

## 2. Materials and Methods

As human sensory perception is an essential indicator of skin texture assessment [6], we used both physical measurements of coarseness and questionnaires which quantified subjects' perceptions in conducting assessments. Skin roughness is related to the aging of the stratum corneum [7]. This is reflected in the fact that the skin surface's structure is often used to evaluate the efficacy of nutrition or cosmetics in creating glossier skin or reducing wrinkles [8–10]. In addition, many studies used examinations of skin hydration and melanin levels to measure skin health and the efficacy of active ingredients used in combating skin aging [11]. This study has been approved by the Research Ethics Committee of the National Cheng Kung University, Tainan, Taiwan (approval no. 103-111-2).

### 2.1. Participants

After we provided written and verbal explanations concerning the details of this study, women who provided signed informed consent were included in this prospective trial. Women with systemic diseases, allergies, contact diseases, or atopic dermatitis were excluded. This study enrolled 80 women, with a mean age of 19.6  ±  0.5 years. Participants were allocated to four groups: an experimental group who received white powder application and facial threading, a negative control group who received white powder application but without facial threading, a positive control group who received exfoliating treatment, and another positive control group who had their faces shaved. Each of the four groups included 20 participants. No whitening, moisturizing, exfoliating, nor medical cosmetic treatments were allowed 4 weeks prior to the treatment period.

### 2.2. Experiment Design

**Figure 1:**
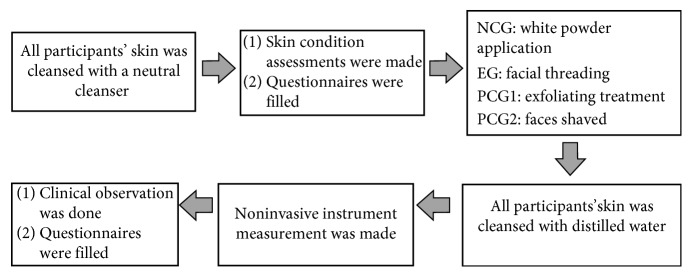
Experiment design.

This study employed questionnaires filled out by participants, clinical observation, and noninvasive instrument measurement under controlled conditions (room temperature 24 ± 2°C and relative humidity 50 ± 5%) [12, 13]. Firstly, all participants' skin was cleansed with a neutral cleanser. Thirty minutes later, skin condition assessments were made. Then, the four groups received their allocated treatments, respectively. After the allocated treatment, all subjects' skin was cleansed with distilled water, with no cosmetics applied. The participants' skin was examined again 30 minutes later (Figure 1).

### 2.3. Allocated Treatments

Participants in the negative control group (NCG) were treated with white powder application but without threading.

**Figure 2:**
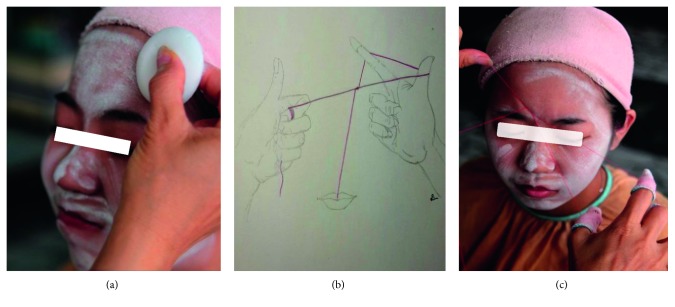
Traditional facial threading illustration. (a) White powder is applied to the entire face. (b) Three-point facial threading technique. (c) Actual process of traditional three-point facial threading.

Participants in the experimental group (EG) received facial threading, and facial threading experts performed three-point facial threading on the participants. Prior to performing facial threading, they applied a layer of white powder on the subjects' faces (Figure 2(a)). The ingredient of the white powder was calcium carbonate, which increased the friction between the skin and the yarn. The person performing facial threading would hold one end of the yarn in her mouth, which served as a fulcrum. She used her left hand to pull the yarn, and the right hand winded the string so that the upper and lower strands formed an angle (Figure 2(b)). She then used both hands to repeatedly drag the thread over the skin. The intertwined threads removed vellus hairs from the participant's face (Figure 2(c)).

An exfoliating cream was used on participants in positive control group 1 (PCG1). After applying the exfoliating cream (from Yangge Co. Ltd., Taiwan; ingredients: carbopol, trimethylglycine, Hibamata, perfumery, and propyl-4-hydroxybenzoate), old keratin was removed from the skin through the friction created by the fingers on the face. This was considered a method of physical exfoliating.

The group who received shaving was positive control group 2 (PCG2). A single professionally trained beautician used a safety razor and gently removed the fine hairs on subjects' faces (except eyebrows or eyelashes).

### 2.4. Noninvasive Instrument Measurements

Because of substantial differences in hydration, pigmentation, and roughness on various areas of the facial skin [14], we chose a fixed area (2 × 2 cm) on the forehead, left cheek, and right mouth corner. Each area was examined three times, respectively, and the mean value was used for analysis.

We utilized a 3D skin surface roughness tester (assesses the Skin Macro Relief by Replica and Oblique Lighting) Skin Visioscan VC98 and Video Digitizer VD300 (CK, Germany) to measure skin roughness. Through software processing and analysis, we obtained a three-dimensional image of the skin texture, as well as roughness measures.

This study used a multifunctional skin assessment system (Cutometer MPA 580, Courage + Khazaka Electronic, Cologne, Germany) and connected a skin hydration exam probe (Corneometer CM 825) to ensure the skin surface's conductivity [15, 16]. In addition, a melanin-erythema index indicator probe was connected (narrowband spectrophotometry; Mexameter MX 18, Courage + Khazaka Electronic, Cologne, Germany) in order to measure skin color through melanin and erythema counts [17, 18]. An infrared wavelength of 880  ± 10  nm, a red-light wavelength of 660 ± 3 nm, and a green-light wavelength of 568 ± 3 nm were used for measurement.

### 2.5. Participant-Rated Questionnaire

The participant-rated questionnaire was composed of the following four sections: (1) An assessment of the participants' skin quality was done. Global skin-type self-evaluation (dry, normal, oily, or mixed skin) was performed by a questionnaire. (2) Prior to allocated treatment, they were asked to mark, based on a spectrum, the darkness or lightness of their skin color, as well as the texture. (3) After treatment, participants were asked to reexamine the darkness or lightness of their skin, as well as the texture, on a spectrum. (4) We held a semistructured interview about their psychological feelings during treatment (for example, whether it was comfortable, hot, pricking, tight, no feeling, or others).

The participant-rated questionnaire employed a semantic differential scale [19] and obtained the sense of feel for participants before and after treatment on a five-point Likert scale. The change in skin color (extremely dark skin is 1 point, while extremely light skin is 5 points) as well as other indicators (extremely rough skin is measured as 1, and extremely smooth skin is measured as 5) was also rated.

### 2.6. Image Analysis

One participant in the facial threading group was randomly selected to receive macro photography examinations before and after the treatment. The same photography lighting setting was used. We used a CANON EOS 550D camera and a high-resolution lens CANON EF 24–70 mm f/2.8L USMII, as well as a SIGMA 105 mm f/2.8 lens (all manufactured in Japan). For the color space, we chose the RGB model. We did not utilize color correction or a spatial filter as we desired to preserve the genuineness of the image.

### 2.7. Statistical Analysis

We used the SPSS 17 software (IBM SPSS Inc., Chicago) for data analysis. The two-tailed paired *t*-test was used to examine whether the skin quality changed after the allocated treatment. We utilized the one-way analysis of variance (ANOVA) test with different skin treatment methods (dry mask, facial threading, exfoliating, and shaving) as independent variables and the skin condition measures as the dependent variable. This study explored whether there were significant differences in skin quality between groups. Finally, multiple comparative analysis (Scheffé's method) was performed using the Tukey test to further explore whether or not differences existed between groups. A *P* value <0.05 was considered significant. Continuous data are presented as mean ± SD unless otherwise stated.

## 3. Results

Each of the four groups included 20 participants. The combination skin type was the most common type among participants, accounting for 80%, 70%, 60%, and 80% of the NCG, EG, PCG1, and PCG2, respectively.

### 3.1. Skin Texture Examination

#### 3.1.1. Forehead

**Figure 3:**
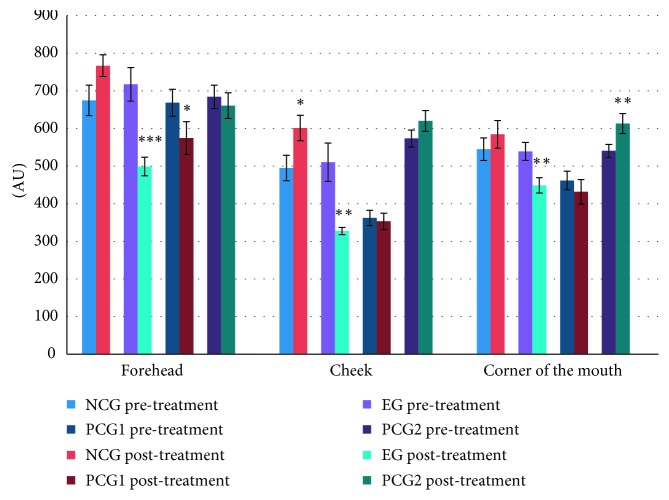
Pre- and posttreatment skin roughness comparison of the forehead, cheek, and corner of the mouth for each group. *n* = 20; ^*∗*^*P* < 0.05; ^*∗∗*^*P* < 0.01; ^*∗∗∗*^*P* < 0.001; NCG: negative control group; EG: experimental group; PCG1: positive control group 1; PCG2: positive control group 2. The bars represent standard deviation. AU: arbitrary unit.

**Figure 4:**
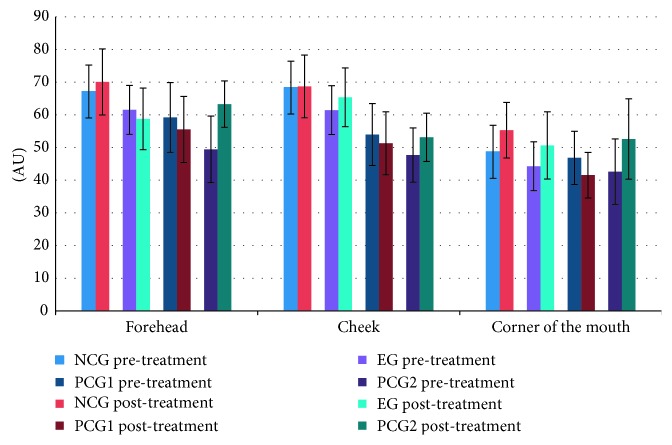
Pre- and posttreatment skin hydration comparison of the forehead, cheek, and corner of the mouth for each group. *n* = 20; ^*∗*^*P* < 0.05; ^*∗∗*^*P* < 0.01; ^*∗∗∗*^*P* < 0.001; NCG: negative control group; EG: experimental group; PCG1: positive control group 1; PCG2: positive control group 2. The bars represent standard deviation. AU: arbitrary unit.

After the allocated treatment, skin roughness indices significantly dropped by 30.4% (*P* < 0.001) and 14.0% (*P*=0.023) in the EG and PCG1, respectively. No significant differences were detected in the NCG and PCG2 (Figure 3). There were no significant differences for changes in hydration indices after treatment in all four groups (Figure 4). No significant changes in the melanin and erythema after allocated treatments were detected in the four groups (data not displayed).

The posttreatment comparisons showed forehead skin roughness indices of the EG approached those of PCG1 and were clearly lower than those of the NCG and PCG2 (Table 1).

#### 3.1.2. Cheek

After the allocated treatment, the skin roughness indices of the cheek skin of the EG significantly decreased by 35.9% (*P*=0.004) but increased by 21.5% in the NCG (*P*=0.05). By contrast, no significant changes were found in PCG1 and PCG2 (Figure 3). There were no significant differences for changes in hydration indices after treatment in all four groups (Figure 4). No significant changes in the melanin, erythema, and hydration indices after allocated treatments were detected in the four groups (data not displayed).

In posttreatment comparisons, the cheek skin roughness indices of the EG and PCG1 were distinctly lower than those of the NCG and PCG2 (Table 1). A significant difference was shown for the four groups with respect to skin hydration indices (*P*=0.003). In addition, the indices of the NCG were clearly higher than those of PCG1 and PCG2 (*P*=0.007 and 0.006, respectively). However, no significant differences in the skin hydration levels were shown between the EG and the other three groups (Table 2). In addition, there were no significant postexperiment differences in melanin and erythema for the four groups.

#### 3.1.3. Corner of the Mouth

In pre- and posttreatment comparisons, the skin roughness indices of the mouth corner skin of the EG significantly decreased by 16.7% (*P*=0.004) but significantly increased by 13.4% in PCG2 (*P*=0.006) (Figure 3). No significant differences in hydration, melanin, and erythema for the 4 groups were found.

After the treatment, the skin roughness indices of the mouth corner of the EG and PCG1 were significantly lower than those of the NCG and PCG2 (Table 1). There were no significant differences in forehead skin hydration, melanin, and erythema indices across the 4 groups.

### 3.2. Participant-Rated Questionnaire

**Figure 5:**
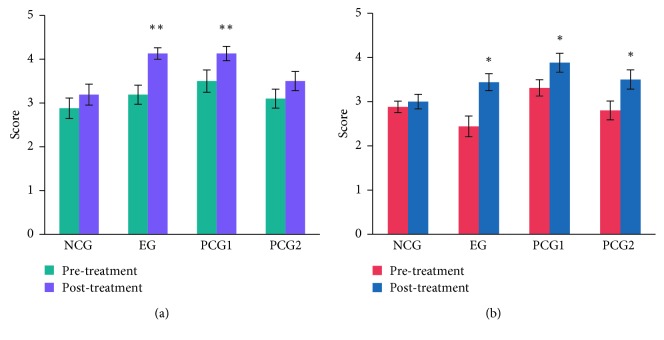
Participant-rated (a) skin touch sense and (b) skin color improvement for the four groups. ^*∗*^*P* < 0.05; ^*∗∗*^*P* < 0.01; NCG: negative control group; EG: experimental group; PCG1: positive control group 1; PCG2: positive control group 2.

The pre- and posttreatment comparisons showed improvement in the participant-rated skin touch sense and skin color for the EG and PCG1 (Figures 5(a) and 5(b)). PCG2 showed significant improvement with respect to skin color, but not in touch sense.

Skin pricking sense was found in the EG as well as PCG1 and PCG2. In the NCG, 100% of subjects (20 people) had no particular sensation. In the EG, 50% of subjects (10 people) felt slight tingling, and the other 50% (10 people) felt extreme tingling. In PCG1, 25% of the study subjects (5 people) felt comfortable, 25% (5 people) felt tightness, 20% (4 people) felt tingling, and 30% (6 people) felt no particular sensation. In PCG2, 85% of subjects (17 people) had no particular sensation, while 15% (3) felt light pricking.

### 3.3. Image Analysis

**Figure 6:**
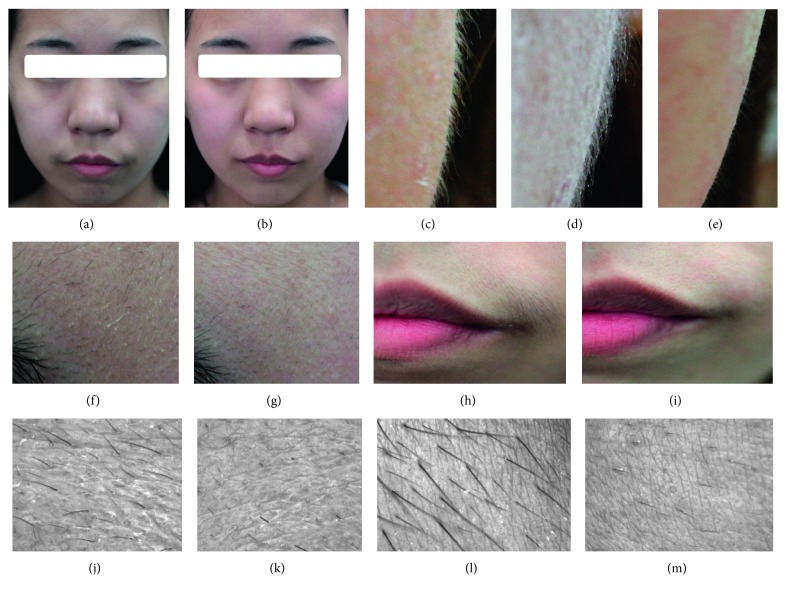
Comparison of fine lines before and after facial threading. Entire face before (a) and after (b) threading. Right cheek before threading (c), after application of a layer of white powder and before threading (d), and after threading (e). Digital image of the forehead skin before (f) and after (g) threading. Digital image of the corner of the mouth before (h) and after (i) threading. 3D image of the forehead skin before (j) and after (k) threading. 3D image of the corner of the mouth before (l) and after (m) threading. 3D images were photographed with Skin Visioscan® VC98 and Video Digitizer VD300.

As shown in Figure 6, the fine lines in various areas of the face became sparser and less conspicuous after facial threading in the EG. Also, the skin texture and appearance were shinier and smoother after facial threading.

## 4. Discussion

This study found facial threading improves the skin coarseness in various parts of the face (Figure 3). In addition, posttreatment comparisons between the four groups for skin coarseness indices showed the EG and PCG1 had lower coarseness compared to the NCG and PCG2 (Table 1), and the differences were significant. This allowed us to observe traditional facial threading was similar in effectiveness to exfoliation.

Pre/postexperiment comparisons for various areas of facial skin of the EG showed no significant differences (Figure 4). Also, postexperiment comparisons of hydration indices of each group showed PCG1 and PCG2 had cheek skin hydration levels clearly lower than those of the NCG (Table 2). The results of this trial indicated relatively stable results of facial threading on skin hydration levels. There were also no significant differences in melanin and erythema indices for all groups in pre- and posttreatment comparisons.

After the facial threading treatment, EG subjects' self-appraised skin touch sense improvement was a level higher (Figure 5(a)), which was consistent with instrument measurement results of skin roughness (Figure 3). However, with respect to skin color improvement, sensory assessments did not support the instrument test results for the EG, PCG1, and PCG2.

Furthermore, according to the clinical observation of the image analysis, vellus hairs became sparser on various parts of the face after facial threading for the EG (Figure 6). This made skin much brighter. Also, the removal of keratin clearly made the skin smoother and brighter, and the tactile sense of skin quality improvement could indirectly create a greater feeling of skin color improvement in participants. Therefore, we could not rule out the possibility that participants in the EG, PCG1, and PCG2 felt subjective improvement in skin color because of multiple influences of visual and tactile senses. Perhaps, this explained the traditional common understanding held by Taiwanese women that facial threading made the skin whiter.

On the contrary, as the vellus hair removal technique in facial threading relied on the external force exerted on the skin, subjects felt a pricking sensation, which could cause concerns about irritation [20]. In future studies, we will seek to assess the possibility of traditional facial threading leading to irritation as well as measures for dealing with such a reaction.

## Figures and Tables

**Table 1:** Changes in the skin roughness indices of the forehead, cheek, and corner of the mouth of the negative control group (NCG), experimental group (EG), and positive control groups 1 and 2 (PCG1 and PCG2).

	*F*	*P*	Post hoc tests				*P*
Forehead	12.058	<0.001	NCG	⟵⟶	EG	▼	<0.001
			NCG	⟵⟶	PCG1	▼	<0.001
			EG	⟵⟶	PCG2	△	<0.003

Cheek	42.868	<0.001	NCG	⟵⟶	EG	▼	<0.001
			NCG	⟵⟶	PCG1	▼	<0.001
			EG	⟵⟶	PCG2	△	<0.001
			PCG1	⟵⟶	PCG2	△	<0.001

Corner of the mouth	10.783	<0.001	NCG	⟵⟶	EG	▼	<0.009
			NCG	⟵⟶	PCG1	▼	<0.003
			EG	⟵⟶	PCG2	△	<0.001
			PCG1	⟵⟶	PCG2	△	<0.001

Decreases (▼) show relatively low skin roughness values, while increases (△) indicate high skin roughness values.

**Table 2:** Summary of variation in the forehead cheek and corner of mouth skin hydration indices of the NCG, EG, and PCG.

	*F*	*P*	Post hoc tests				*P*
Cheek	5.241	0.003	NCG1	⟵⟶	PCG1	▼	0.007
			NCG1	⟵⟶	PCG2	▼	0.006

Decreases (▼) show relatively low skin hydration values.

## Data Availability

The data used to support the findings of this study are available from the corresponding author upon request.
